# Supplementary material for: Comparative transcriptomic rhythms in the mouse and human prefrontal cortex
Source: Front Neurosci. 2025 Jan 13;18:1524615. doi: 10.3389/fnins.2024.1524615 (PMC11769989; doi:10.3389/fnins.2024.1524615)
Supplement: Supplementary file 1 [file Data_Sheet_1.DOCX]

Supplementary Materials and Methods

RNAscope *in situ* hybridization was performed on fresh frozen frontal cortical tissue sections. Adult (~20 weeks) C57BL/6J mice were sacrificed via cervical dislocation at the peak time of expression, as determined from RNA sequencing data, and 12 hours later (ZT6 and ZT18) (n=3/timepoint; 2F, 1M). Serial 14µm sections were cut on a cryostat to isolate sections containing the medial prefrontal cortex (mPFC). mPFC containing sections were mounted onto slides and processed according to the RNAscope Multiplex Fluorescent Reagent Kit v2 protocol (UM 323100; ACD Bio, Newark, CA, USA) using the *Per2* probe (RNAscope Probe- Mm-Per-C2, cat# 454521-C2) and counterstained with DAPI. Slides were imaged at 40x magnification using an Olympus SLIDEVIEW VS200 research slide scanner. The mPFC, containing the prelimbic region, was defined according to (George Paxinos and Franklin, 2001). *Per2*-positive puncta were counted using QuPath software by an experimenter blinded to condition. Cells were defined by the presence of a DAPI stained nucleus. Data was analyzed using an unpaired T-test (GraphPad Prism version 9) and are presented as mean±SEM.

Supplementary Figures

Figure S1: Diurnal rhythms in *Per2* expression in the mouse mPFC

RNAscope was used to confirm the presence of diurnal rhythms in the core clock gene *Per2* using an additional cohort of mice. (A) Representative images of RNAscope images taken at ZT6 (left) and ZT18 (right). Images of the sections taken at 40x with the region of interest (mPFC) outlined in red are shown on top. Manually zoomed in images of the region of interest are shown on the bottom. The number of puncta within each cell (red dashed line around the nucleus) was quantified. Blue=DAPI, Yellow=*Per2* (B) The average number of *Per2* puncta per cell is significantly higher at ZT18 than ZT6 (Unpaired T-test: t(4)=4.851; p=0.008). Data presented as mean ±SEM. **=p<0.01. mPFC=medial prefrontal cortex, ZT=Zeitgeber time

Figure S2: Greatest overlap in rhythmic transcripts between the mPFC and ACC in females at a more stringent cutoff

Venn diagrams depicting rhythmic transcript overlap (p<0.01) between the mouse mPFC and human PFC subregions. mPFC=medial prefrontal cortex, DLPFC=dorsolateral prefrontal cortex, ACC=anterior cingulate cortex

Figure S3: Greater conservation of rhythms between the mouse mPFC and the baboon PFC

(A) Venn diagrams depicting the overlap of rhythmic transcripts between the baboon PFC and mouse mPFC in males. Approximately 20% of transcripts that are rhythmic in the mouse mPFC are also rhythmic in the baboon PFC. (B) Overlap of the top pathways (identified by Ingenuity Pathway Analysis) enriched for rhythmic transcripts in the mouse mPFC and baboon PFC. Nearly half of the pathways (9) are significantly enriched for rhythmic transcripts in both the mouse mPFC and baboon PFC. Baboon data from (Mure et al., 2018). mPFC=medial prefrontal cortex

Supplementary Tables

Table S1: Total reads and read counts (total reads x alignment rate) for mouse samples

Table S2: Rhythmic transcripts (p<0.05) in mouse mPFC with sexes combined

Table S3: Rhythmic transcripts (p<0.05) in mouse mPFC in females

Table S4: Rhythmic transcripts (p<0.05) in mouse mPFC in males

Table S5: Rhythmic transcripts (p<0.05) in DLPFC in females

Table S6: Rhythmic transcripts (p<0.05) in DLPFC in males

Table S7: Rhythmic transcripts (p<0.05) in ACC in females

Table S8: Rhythmic transcripts (p<0.05) in ACC in males

Supplementary Figures

A.


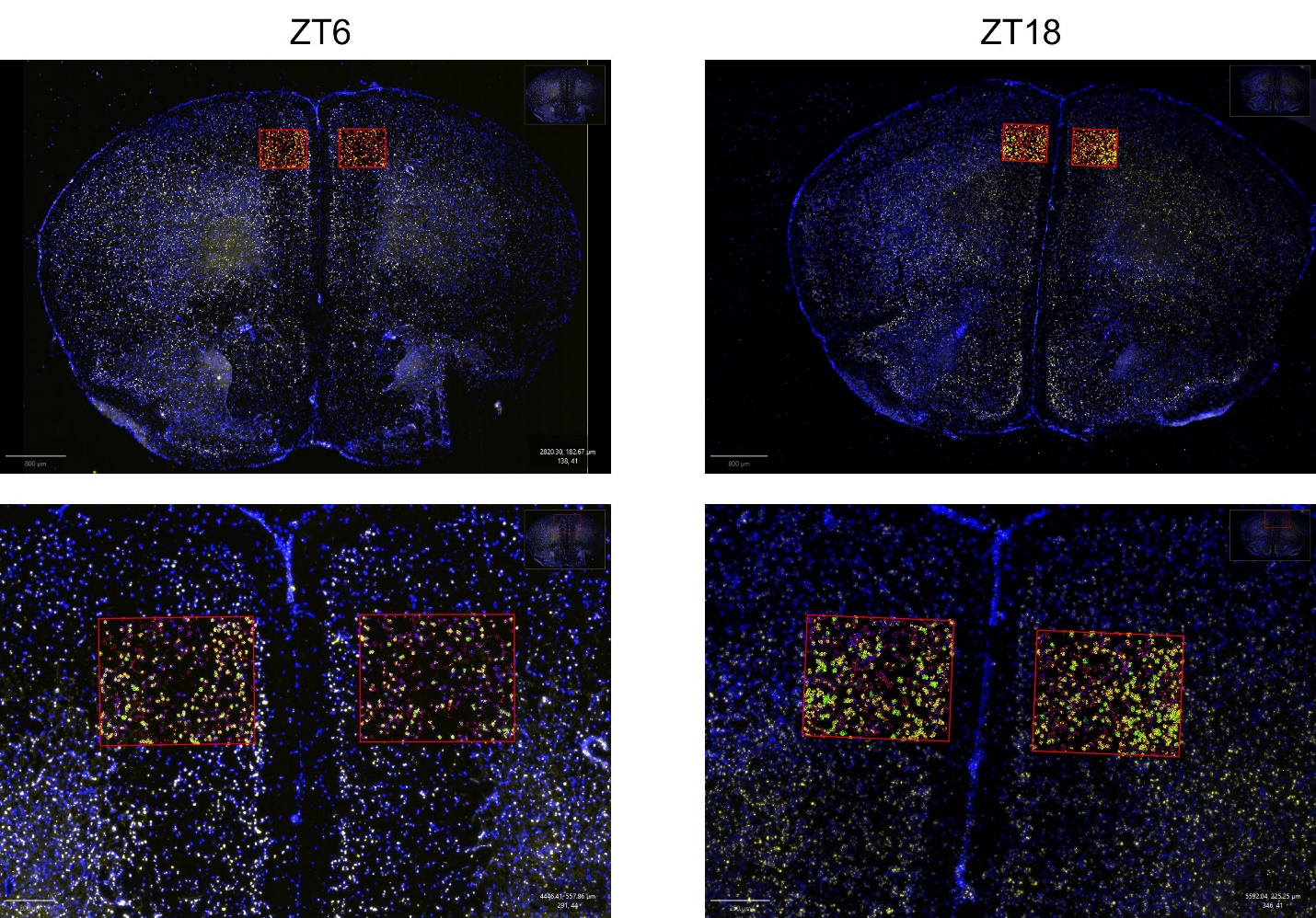


B.


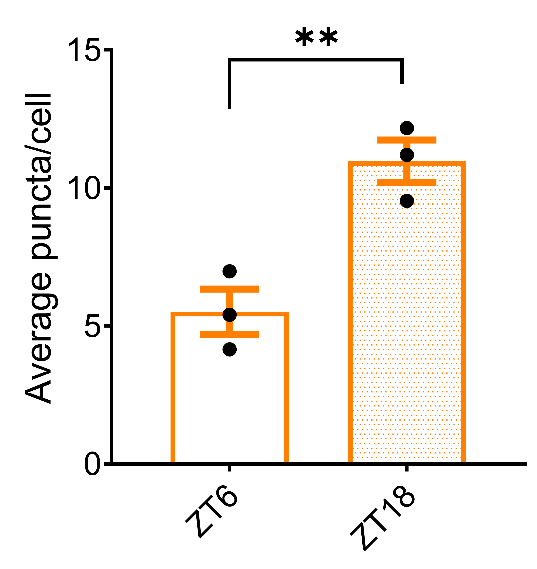


Figure S1


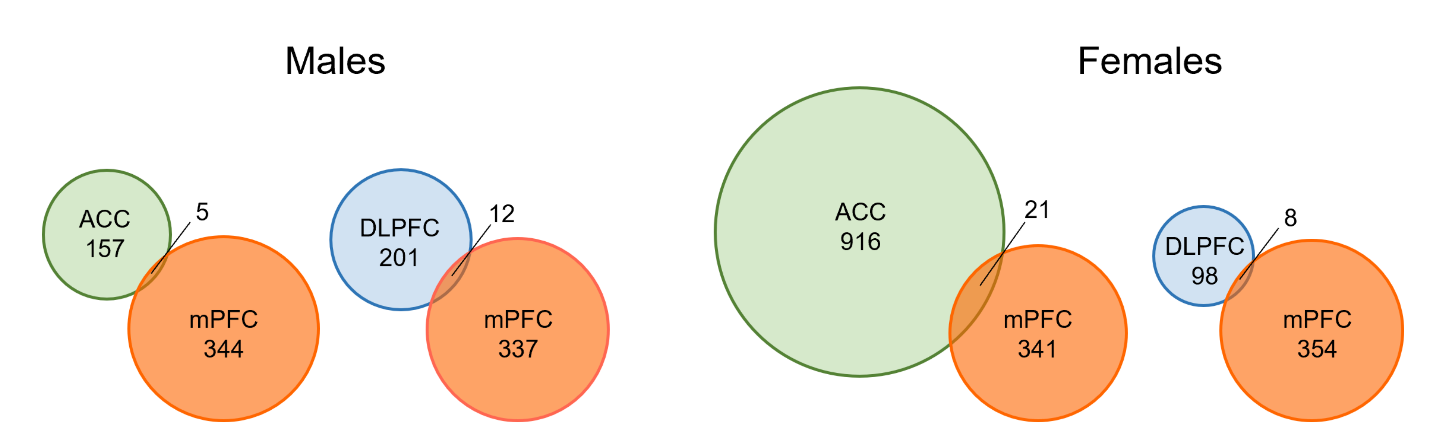


Figure S2


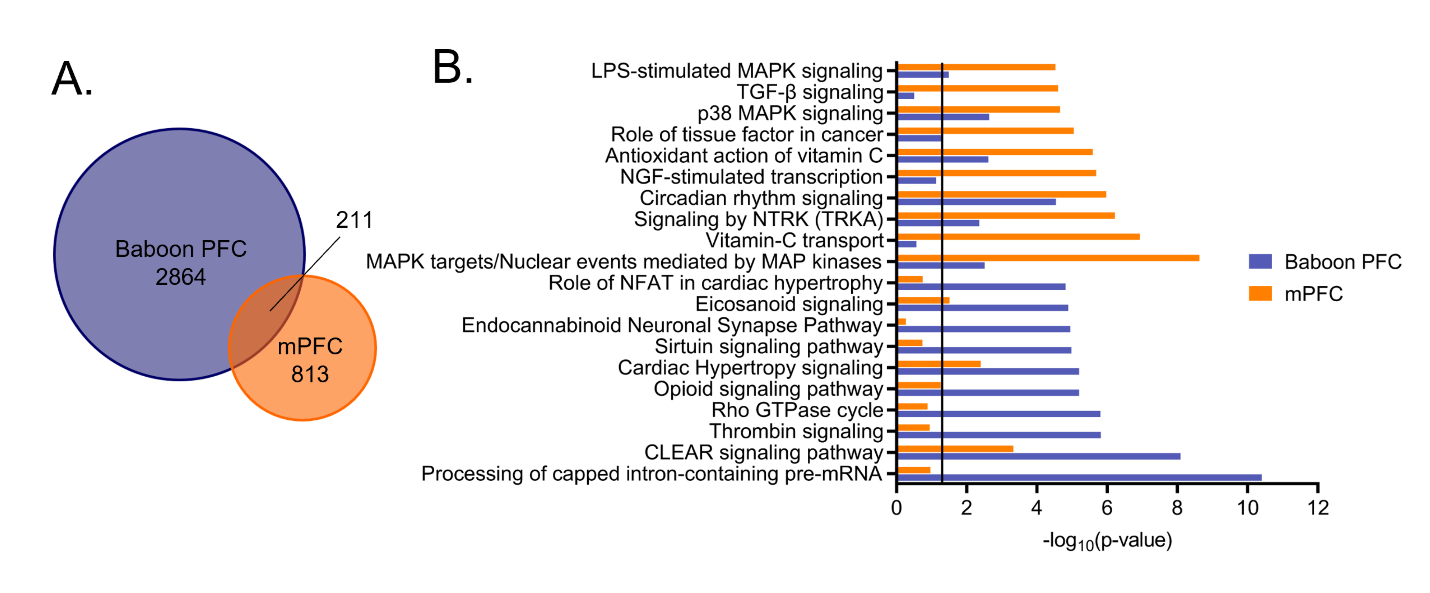


Figure S3

References

George Paxinos, K., and Franklin, B. J. (2001). The mouse brain in stereotaxic coordinates. *Academic Press.[Google Scholar]*.

Mure, L. S., Le, H. D., Benegiamo, G., Chang, M. W., Rios, L., Jillani, N., et al. (2018). Diurnal transcriptome atlas of a primate across major neural and peripheral tissues. *Science* 359, eaao0318. doi: 10.1126/science.aao0318
