# Supplementary figures and images for: Comparative transcriptomic rhythms in the mouse and human prefrontal cortex
Source: Front Neurosci. 2025 Jan 13;18:1524615. doi: 10.3389/fnins.2024.1524615 (PMC11769989; doi:10.3389/fnins.2024.1524615)

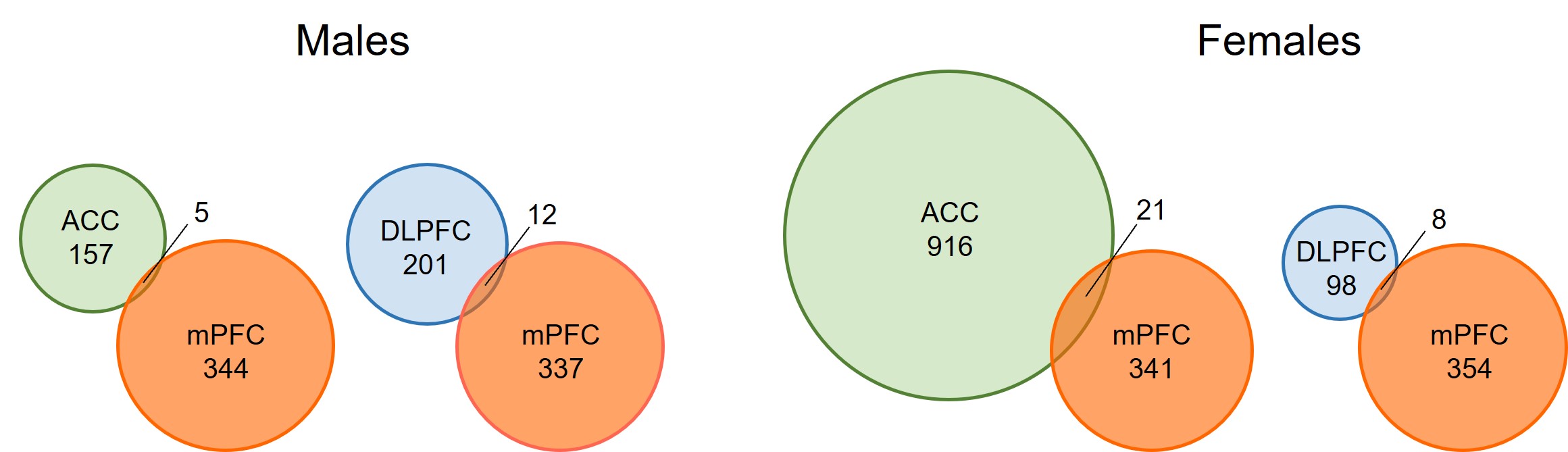

Supplement: Supplementary file 3 [file Image_2.JPEG]

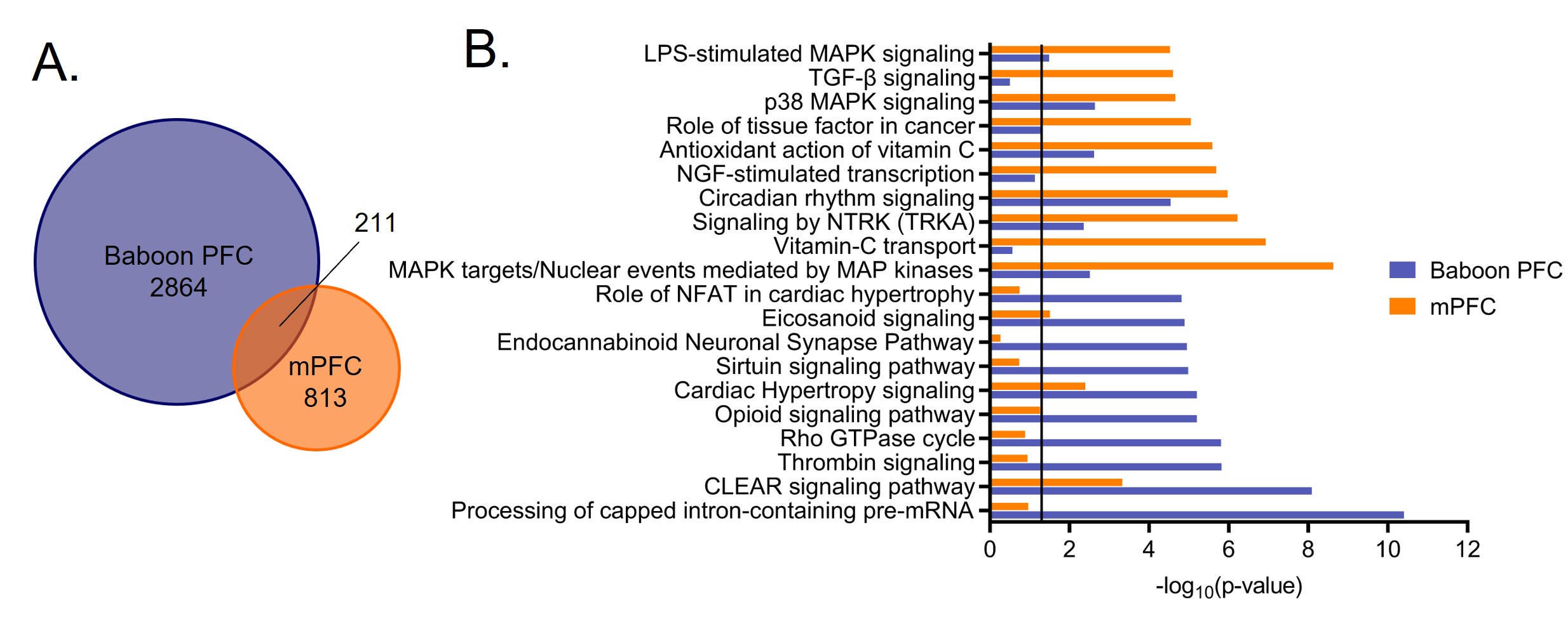

Supplement: Supplementary file 4 [file Image_3.JPEG]
